# Efficacy of P_11_-4 for the treatment of initial buccal caries: a randomized clinical trial

**DOI:** 10.1038/s41598-020-77057-3

**Published:** 2020-11-19

**Authors:** Paulina Sedlakova Kondelova, Alaa Mannaa, Claudine Bommer, Marwa Abdelaziz, Laurent Daeniker, Enrico di Bella, Ivo Krejci

**Affiliations:** 1 University of Geneva, University Dental Clinics CUMD, rue Michel-Servet 1, 1211 Geneva 4, Switzerland; 2 Division of Operative Dentistry, Department of Restorative Dentistry, Faculty of Dentistry, King Abdulaziz University, P.O. Box 80209, Jeddah, 21589 Kingdom of Saudi Arabia; 3 Credentis AG, Dorfstrasse 69, 5210 Windisch, Switzerland; 4 Department of Political Sciences, University of Genova, P.le E. Brignole 3a, 16126 Genova, Italy

**Keywords:** Dental caries, Biomineralization

## Abstract

To investigate the safety and efficacy of Self-Assembling Peptide P_11_-4 (SAP P_11_-4) compared to placebo or fluoride varnish (FV), a randomized, controlled, blinded, split-mouth study with sequential design was conducted. Subjects presenting two teeth with White-Spot-Lesions (WSLs) were included and teeth were randomly assigned to test or control. Control received placebo at baseline (D0) and test SAP P_11_-4, all received FV at Day 90 (D90). Standardized photographs were taken at each visit, and WSL size changes were morphometrically assessed. Hierarchical Linear Modelling, considering paired and sequential design, was used to test four hypotheses. SAP P_11_-4 lesions (test, D90–D0) showed significant WSL size reduction compared to placebo (control, D90–D0; p = 0.008) or FV (control, D180–D90; p = 0.001). Combination of SAP P_11_-4 and delayed FV after 90 days (test, D180–D0), showed a significant difference compared to FV alone (control D270–D90; p = 0.003). No significant difference on FV efficacy was found when SAP P_11_-4 was applied 3 months before FV (test D270–D90; control D270–D90, p = 0.70). SAP P_11_-4 treatment resulted in superior caries regression compared to either placebo or FV, and FV efficacy seems not to be affected by SAP P_11_-4. SAP P_11_-4 was found to be a safe and effective WSL treatment.

## Introduction

Modern dentistry has witnessed a paradigm shift in dental caries management. The medical model, which focuses on caries prevention, reduction and reversal of incipient lesions, has gradually replaced the traditional interventional model known as the “drill and fill model”^1^. Over the past decades, fluoride, in various application forms, has been the predominant tool for caries prevention, with sufficient supporting evidence for its efficacy and safety^2,3^. Its widespread use is generally considered as the main cause for dental caries reduction^4^. However, shortcomings of the present fluoride therapies have been reported, including limited efficacy in some subjects, a caries reduction plateau at the population level^5^, and ineffectiveness in caries already manifested as white spots^6^. Subsequently, new remineralization therapies have been called for^7^ and are being developed to close this treatment gap by either adding or enhancing the effect of fluoride or replacing them, also for subjects reluctant to use fluoride^4^.

Breakthroughs in biomimetic remineralization have enabled the oral remineralization process, driven by saliva, beyond its normal level by providing additional nucleation sites for the crystallization of calcium phosphate^8^. Recently, a rationally designed monomeric self-assembling peptide P_11_-4 (SAP P_11_-4) solution^9^, which is able to diffuse into the subsurface carious lesions and self-assembles into a 3D biomimetic scaffold within, can guide the remineralization process^10,11^. SAP P_11_-4 has been shown to diffuse into the body of the carious lesion, and supporting de novo hydroxyapatite formation below the porous mineralized surface^11–14^. A first in man clinical trial has shown that SAP P_11_-4 is safe for human use and can reverse caries progression, significantly reducing the size of buccal white spot lesions as assessed by a visual analogue scale^15^. Another early clinical trial has shown that, when SAP P_11_-4 is applied on proximal carious surfaces, it leads to an arrest or even regression of caries in 85% of cases as judged by standardized clinical radiographs^16^. Recent randomized gold-standard controlled clinical trials have shown SAP P_11_-4 combined with fluoride varnish or a Self-Assembling Peptide Matrix Gel to be superior for the treatment of early occlusal caries compared to fluoride varnish alone^17,18^. The combination treatment inactivated 80–100% of the carious lesions and caused regression into a lower ICDAS-II class in up to 30% of cases. In contrast, in the control groups solely receiving fluoride varnish, only 35% of the carious lesions were inactivated, and 6% regressed to smaller ICDAS-II class^17^ and 20% needed restorative treatment during the 12 months study period^18^.

The aims of the present randomized and quadruple-blinded clinical split-mouth trial with the unique sequential study design allowing in a single trial to investigate the following objectives: (1) the efficacy of SAP P_11_-4 in the treatment of incipient buccal carious lesions in a comparative study, (2) compare, for the first time, the efficacy of SAP P_11_-4 with a placebo and with a fluoride varnish treatment alone, (3) assess the additional benefit of SAP P_11_-4 in the combined treatment with fluoride varnish in comparison to fluoride varnish alone, and (4) evaluate the influence of SAP P_11_-4 on the efficacy of fluoride varnish.

## Results

The study protocol was approved by the Commission d’Ethique of the Hospitaux Universitaires de Geneve (12-275, approval date: 22/05/2013) and registered in clinicaltrials.gov (NCT02020681, registration date: 19/12/2013) prior to study start.

### Subjects

Overall, 44 subjects (26 female, average 27.1 years) were enrolled from the daily practice and the student body of the University of Geneva between November 2013 (first patient’s first visit) and January 2016 (last patient’s last visit). All subjects signed informed consent prior to any study-related procedures. Subject’s as well as study lesion’s baseline characteristics are presented in Table 1. Initial characteristics of the study lesions were recorded by two clinicians and were comparable for the test and control arms regarding the Clinical Index White Spot Lesion^19^, lesion’s appearance and texture, and lesion activity. Lesions of subjects who had an orthodontic treatment and finished it more than one year before study inclusion were not classified as lesions originating from orthodontic treatment. Notably, white spot lesions originating from orthodontic treatment, known to have a partial remineralization potential without any treatment^20,21^ were absent in the present study. In total, 44 tooth-pairs (one per subject) were randomly assigned to either test or control treatment arm and analysed. One subject was lost to follow-up after the Day 30 (D30) visit due to relocation, and 3 subjects were lost to follow-up after Day 90 (D90) due to unknown reasons (Consort chart Supplementary Fig. S1).

**Table 1:** Baseline data of subjects and white spot lesions.

Parameter	Study population	
Number of subjects	44	
Age	27.1 (15–39) years	
Sex	18 (41%) male 26 (59%) females	
Ethnicity	Caucasian	36 (82%)
	Arabian	3 (7%)
	African	3 (7%)
	Indian	2 (4%)
DMFT	4.4 ± 5.1 (0–21)	
Oral hygiene	Poor	0
	Unsatisfactory	3 (7%)
	Sufficient	5 (11%)
	Good	11 (25%)
	Excellent	25 (57%)

	Test lesions (n = 44)	Control lesion (n = 44)
Plaque index	0.20 ± 0.25 (0–1.2)	
Gingival index	0.08 ± 0.12 (0–1.2)	
Clinical index white spot lesion*		
0	0 (0%)	0 (0%)
1	2 (4%)	1 (2%)
2	7 (16%)	10 (23%)
3	18 (41%)	19 (43%)
4	17 (39%)	14 (32%)
**Lesion appearance**		
Shiny	16 (36%)	17 (39%)
Opaque	28 (64%)	27 (61%)
**Lesion texture**		
Smooth/hard	34 (77%)	34 (77%)
Rough/soft	10 (23%)	10 (23%)
**Lesion location/origin**		
Far from sulcus	1 (2%)	2 (4%)
Close to sulcus	43 (98%)	42 (96%)
Post-orthodontic	0 (0%)	0 (0%)
**Nyvad score**		
Active (Score 1, 2)	25 (57%)	30 (68%)
Inactive (Score 4, 5)	19 (43%)	14 (32%)
**Laser fluorescence**		
Mean (SD)	6.7 ± 5.3	6.5 ± 4.9
Min	2	3
Median	5	5
Max	29	32
Counts (< 10: healthy)	40 (91%)	39 (89%)
Counts (≥ 10: caries)	4 (9%)	5 (11%)
Lesion size (morphometry/pixel)	53′023 ± 70′022	50′787 ± 69′584
Clinical phase (first patient in—last patient out)	November 2013–January 2016	

*0: No visible colour change, 1: Slight white colour change, only visible after air-drying, 2: Slight white colour change with certain marked white areas, 3: White consistent colour change, 4: Distinct white colour change.

### Morphometry

Both test and control lesions had a comparative size at baseline (test arm: 53′023 ± 70′022 pixel; control arm: 50′787 ± 69′584 pixel).

The relative size of the control arm lesions decreased on average by:

− 0.07 ± 0.24 between D0 and D90 (placebo, 11/43 decreasing by ≥ − 0.2; 5/43 increasing by ≥ + 0.2),
− 0.03 ± 0.13 between D90 and D180 (effect of fluoride varnish for 90 days, whereby 4/40 decreased by ≥ − 0.2 and 2/40 increased by ≥ + 0.2),
− 0.04 ± 0.19 between D90 and D270 (effect of fluoride varnish for 180 days, whereby 7/39 decreased by ≥ − 0.2 and 2/39 increased by ≥ + 0.2).

The relative size of the test arm lesions decreased on average by:

− 0.19 ± 0.25 between D0 and D90 (SAP P_11_-4, with 17/41 decreasing by ≥ − 0.2; 0/41 increasing by ≥ + 0.2),
− 0.20 ± 0.28 between D0 and D180 (SAP P_11_-4 followed by a 3 months delayed fluoride varnish, whereby 20/39 decreased by ≥ − 0.2 and 1/39 increased by ≥ + 0.2),
− 0.06 ± 0.18 between D90 and D270 (fluoride varnish with prior SAP P_11_-4, whereby 6/38 decreased by ≥ − 0.2 and 2/38 increased by ≥ + 0.2).

(Table 2, Box plots see Supplementary Fig. S2, Supplementary Table 1).

**Table 2:** Morphometric Assessment of White Spot Lesions and Comparisons between groups as observed and estimated using hierarchical linear modelling (HLM).

Comparison (n = 44)	Corresponding time periods	Change in lesion size (SD) (observed values)		HLM (adjusted, estimated values)	p-Value*
		Test group	Control group	Treatment estimate^a^ (SE)	
SAP P_11_-4 vs placebo	Test D90–D0 vs control D90–D0	− 0.19 (0.25)	− 0.07 (0.24)	− 0.14 (0.05)	**0.008**
SAP P_11_-4 vs FV	Test D90–D0 vs control D180–D90	− 0.19 (0.25)	− 0.03 (0.13)	− 0.18 (0.05)	**< 0.001**
SAP P_11_-4 + FV vs FV	Test D180–D0 vs control D270–D90	− 0.20 (0.28)	− 0.04 (0.19)	− 0.17 (0.05)	**0.003**
FV (prior SAP P_11_-4) vs FV	Test D270–D90 vs control D270–D90	− 0.06 (0.18)	− 0.04 (0.19)	− 0.02 (0.04)	0.70

*p-Values indicating statistical significance are marked in bold.

^a^Tested effect: treatment groups with HLM adjusted for gender, race, age, DMFT, WSL clinical index, oral hygiene and plaque index baseline values. Reference is control group.

### Group comparisons

The difference in WSL size for the four group comparisons of interest (Hypotheses H1–H4) was investigated using Hierarchical Linear Modelling (HLM) controlling for confounding factors (Table 2, Supplementary Tables 2–5).

Hypothesis 1 (H1): A statistically significant difference was observed for the change of WSL size treated with SAP P_11_-4 showing a size reduction of − 0.14 compared to WSL treated with placebo (95% CI − 0.04 to − 0.24, p = 0.008).

Hypothesis 2 (H2): A statistically significant difference was observed for the change of WSL size treated with SAP P_11_-4 showing a size reduction of − 0.18 compared to WSL treated with fluoride varnish (95% CI − 0.08 to − 0.27, p < 0.001).

Hypothesis 3 (H3): A statistically significant difference was observed for the change of WSL size treated with SAP P_11_-4 followed by a 3-month delayed fluoride varnish treatment, showing a size reduction of − 0.17 compared to WSL treated fluoride varnish alone (95% CI − 0.06 to − 0.28, p = 0.003), indicating an additional benefit of SAP P_11_-4 in a treatment period of 180 days compared to fluoride varnish alone.

Hypothesis 4 (H4): No statistically significant difference was observed for change of WSL size treated with fluoride varnish after receiving SAP P_11_-4 3 months earlier (estimate of size reduction − 0.02), compared to lesions treated with fluoride varnish that had not received SAP P_11_-4 previously (95% CI − 0.10 to 0.06, p = 0.70).

The results computed with HLM proved to be robust in the sensitivity analysis (Supplementary Tables 6–12).

### VAS progression scores

The result of the evaluation of the progression/regression of the WSL by means of VAS with respect to D0 was as follows:

A. Placebo (control arm: D90–D0) showed an average of − 4.8 ± 13.9; a median of − 2.0 and a minimum–maximum range of − 30 to + 31, indicating overall values just below "0" corresponding to a slight regression;
B. SAP P_11_-4 (test arm: D90–D0) showed an average of − 8.5 ± 12.9, a median of − 5.0 and range of − 36 to + 17 indicating an overall regression of the lesions with individual exceptions;
C. SAP P_11_-4 with delayed fluoride varnish (test arm: D180-D0) showed an average of − 8.8 ± 12.5, median of − 7.0 and range of − 38 to + 17 indicating overall regression after SAP P_11_-4 application followed by a stabilization after fluoride varnish application.

### Global impression of change

The results of the GIOCQ for the meaningful groups were as follows:

A. Placebo (control arm: D90): very much worse: 0; worse: 3; a little worse: 5; unchanged: 14; a little better 12; better: 9; very much better: 0; missing: 1.
B. SAP P_11_-4 (test arm: D90–D0): very much worse: 0; worse: 0; a little worse: 5; unchanged: 14; a little better 13; better: 6; very much better: 3; missing: 3.
C. SAP P_11_-4 with delayed fluoride varnish (test arm: D180): very much worse: 0; worse: 1; a little worse: 2 unchanged: 14; a little better 14; better: 7; very much better: 1; missing: 5.

### Caries diagnostics

Laser Fluorescence values at D0 for test arm lesions were 6.7 ± 5.3 and 6.5 ± 4.9 for the control arm lesions. According to the manufacturer’s instructions, values < 10 are regarded as healthy enamel. At D0, 4/44 test lesions and 5/44 control lesions indicated carious enamel. On D90, 4/43 of the test lesions were assessed to be carious, whereas in the control lesions 3/43 indicated carious enamel. On D180, the number of carious findings increased to 5/40 in the test lesions and remained at 3/40 in the control lesions. On D270, 5/40 of the test lesions and 3/40 control lesions indicated carious enamel (Supplementary Table 13).

### Nyvad caries activity criteria

The results of the Nyvad Caries Activity Criteria for the meaningful groups were as follows (Supplementary Table 14):

A. Placebo (control arm: D90–D0): becoming inactive: 10.
B. SAP P_11_-4 (test arm: D90–D0): becoming inactive: 8.
C. SAP P_11_-4 with delayed fluoride varnish (test arm: D180–D0): becoming inactive: 10.

Plaque index and oral hygiene data are presented in Supplementary Table 15 and 16.

### Safety

No trial nor intervention-related safety concerns were reported.

## Discussion

SAP P_11_-4 has previously been shown to be a successful treatment for early carious lesions (i.e. White Spot Lesions—WSL) that in combination with fluoride varnish or Self-Assembling Peptide Matrix Gel led to regression and inactivation of occlusal caries^17,18^ as well as to regression of proximal lesions, the later assessed on standardised clinical radiographs^22^. Furthermore, it has been shown to positively influence the appearance of buccal white spot lesions^15^. In a recently reported split-mouth study, investigating SAP P_11_-4 in early buccal caries within a private practice setting, morphometry was also used as primary outcome measure similar to the present study^23^. In Broeseler et al. 2020 the fluoride varnish control group showed a stabilisation of the lesion’s size over a duration of a year, whereas the SAP P_11_-4 test group resulted in a size reduction.

The current clinical trial supplied additional information relating to the performance of SAP P_11_-4 to standard clinical care, such as fluoride varnish or wait-and-see (i.e., placebo), as measured by the WSL size. SAP P_11_-4 induced regression of caries and has shown to be both superior to the placebo control (including cleaning and etching of the lesion) and the gold-standard fluoride varnish. SAP P_11_-4 facilitates de novo hydroxyapatite formation throughout the subsurface carious lesion, clinically visible as a decrease in lesion size^10^. In contrast, fluoride ions promote remineralisation only within the top 20 μm of the enamel surface^24^ resulting in stabilisation of the carious lesion as observed morphometrically in the present clinical trial.

The sequential design of the present study allowed for multiple hypothesis to be tested, and it was shown that treatment with SAP P_11_-4 does not impede on the efficacy of fluoride varnish, when SAP P_11_-4 is applied three months before fluoride varnish. The present study cannot rule out an effect of SAP P_11_-4 on fluoride varnish’s efficacy as there maybe absence of evidence which does not proof an absence of effect. However, the present results add evidence to two previously reported SAP P_11_-4 studies on occlusal caries, but as these studies did not include a treatment group solely receiving SAP P_11_-4, inhibitory effects of SAP P_11_-4 on fluoride varnish could not be excluded. It should be noted that the WSLs treated with SAP P_11_-4 in the present clinical trial did not disappear completely, in agreement with previous reports^15,21,25^ and consistent with the fact that the formed hydroxyapatite crystals formed on the SAP P_11_-4 scaffold surface show a fan-type structure not a prismatic arrangement^10^ and thus do not result in the same translucency as natural prismatic enamel. The changes in the white spot lesion should be and were in the present study regarded as a surrogate parameter and not a direct measure of caries or a clinical success parameter. An estimation of clinical success might be the number of WSLs decreasing by > 20% in size and are therefore clearly regressing by visual inspection or the number of buccal lesions progressing in size > 20%, in analogy clearly progressing. For those criteria, SAP P_11_-4 led to 42% of the lesions regressing, while none progressed in size, the placebo group led to 26% regressing and 12% progressing, and the fluoride varnish group led to 10% regressing and 5% progressing. These findings correspond to the assumption that SAP P_11_-4 remineralizes the subsurface body of active carious lesions and has little effect on inactive carious lesions, fluoride varnish closes the mineralized surface and thus inactivates the lesion, and placebo-treated lesions are more dynamic and progress or regress depending on subject behaviour.

The results of the present trial are consistent with previous clinical studies on SAP P_11_-4^15,17,18,21,22^, indicating that SAP P_11_-4 could be a suitable clinical option for the non-invasive treatment of early carious lesions, allowing a more comprehensive and in-depth regression of decayed enamel^7,26^.

As spontaneous enamel regression is generally not observed^27^, except in WSLs originating from orthodontic treatment after debonding^28^, the treatment with SAP P_11_-4 can ultimately facilitate the preservation of the tooth structure and delay or avoid entry into the deadly spiral of restorations^29,30^ allowing potentially a longer lifetime of the tooth.

In the present study, the appearance and progression/regression of WSLs was chosen as the primary parameter, as it resembles the clinical indicator on which clinicians base their treatment decisions^31,32^. This is apparent from the primarily used classification system of caries (ICDAS-II) and clinical radiographs, which rely on visual or radiographic appearance^33^.

Although WSLs seldomly receive dedicated treatment, as they do not pose an imminent clinical problem, the appearance and WSL size is closely followed by clinicians, triggering invasive treatment when significant progression is observed^34^. However, other factors, such as opaqueness as an indicator for activity, add to the treatment decision^35^.

The limitations of the present study are that WSL size is an indirect surrogate measurement of caries, which cannot be directly and quantitatively related to clinical success, and in order to obtain high quality standardized photographs, buccal carious lesions were chosen, of which many were inactive at the study start as evident from the Nyvad Caries Activity Criteria and Laser Fluorescence readings. This led to an effectively smaller sample size with regard to lesion activity, resulting in low laser fluorescence baseline values and preventing meaningful quantitative assessment. In a future study carious lesion activity should be an inclusion criteria as defined by Nyvad Caries Activity Criteria and/or Laser Fluorescence threshold values.

The used sequential study design could also be regarded as a limitation. Yet to avoid biases in a corresponding study with six arms, it would have needed significantly more subjects to be enrolled, resulting in greater human and financial resources as well a longer recruitment time and multiple investigators/sites, which would have required extensive calibration among them. With the HLM analysis, precautions were taken to counteract bias by controlling for confounders. The present design was chosen to obtain as much information as possible with as little downsides as possible.

Lastly, due to the split-mouth study design and ethical implications of giving oral hygiene instructions and fluoride toothpaste at every study visit, the placebo group was not without treatment.

## Conclusion

Treatment of early buccal carious lesions with SAP P_11_-4 led to superior regression of caries decay compared to either placebo or fluoride varnish. Furthermore, it was demonstrated that SAP P_11_-4 did not impede the effectiveness of fluoride varnish, and treatment with SAP P_11_-4 with delayed fluoride varnish was superior to treatment with fluoride varnish alone. Based on current and previous clinical studies, SAP P11-4 may change the daily management of initial carious lesions from a wait-and-see approach to early non-invasive interventions with the intent to regenerating the enamel tissue. Since SAP P11-4 supports the formation of de novo hydroxyapatite crystals deep within and throughout the carious lesion body, it offers the clinician a new, effective, non-aerosol generating and non-invasive treatment option.

## Materials and methods

### Subjects

All clinical procedures were performed at the University of Geneva, University Clinics of Dental Medicine according to the approved study protocol and current versions of ISO 14155, Declaration of Helsinki and Swiss regulations on clinical trials. Subjects presenting with at least two Class V lesions not in need of invasive treatment and not on adjacent teeth were eligible for the study. The main selection criteria were: lesions must be fully visible, assessable and accessible; > 20 teeth and permanent dentition; < 65 years of age; written informed consent before study participation; negative pregnancy test; willing to observe good oral hygiene throughout the study; no adjacent restorations on the study tooth surface; fluoride varnish application ≥ 6 months prior to study; no evidence of tooth erosion; and no concurrent participation in another trial.

### Study design and randomization and treatments

**Figure 1:**
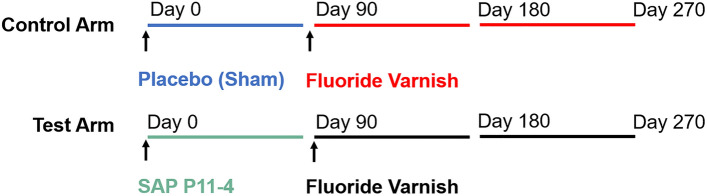
Clinical trial design depicting the recalls and interventions for test and control lesions. Time intervals within the sequential design corresponding to groups in the comparisons are depicted as follows: Placebo (Sham) (blue); fluoride varnish (control arm) (red); SAP P_11_-4 (green); fluoride varnish (test arm) (black).

This randomized, placebo and gold-standard controlled, blinded split-mouth clinical trial with sequential design was designed to investigate the clinical effect of SAP P_11_-4 for the treatment of early buccal carious lesions compared to a sham/placebo treatment, and fluoride varnish, as well as to assess the additional benefit of SAP P_11_-4 in a combination treatment with fluoride varnish in comparison to fluoride varnish alone and to evaluate the influence of SAP P_11_-4 on the effectiveness of fluoride varnish (Fig. 1). The trial was quadruple-blinded (subject, investigator, assessor, statistician). A blinded randomization sequence (defined via higher/lower tooth number) was generated by a third party and kept in numbered, closed envelopes at the investigator site. The computer-generated randomisation sequence of the test and control tooth allocation was kept under lock at third party (sealed envelopes were available at trial site in the case of an emergency) and was provided for unblinding only after morphometric assessment and statistical analysis was performed. Bags with the same numbering as on the envelopes containing the study material were provided to the study centre. Each numbered bag contained one vial of verum and one of placebo (indistinguishable from verum vial). The vials were labelled with "higher tooth number" or "lower tooth number" according to the computer-generated tooth assignment. After subjects met the selection criteria, a sequential envelope was drawn providing the tooth allocation for the treatments of the two teeth per study participant to either the test or control arm (simple randomization; 1:1). At baseline (D0), test lesions received SAP P_11_-4 (50 µL Curodont Repair, credentis ag), and control lesions received placebo (50 µL). All study teeth received fluoride varnish according to manufacturer’s instructions (~ 25 µL/tooth; 22,600 ppm fluoride, Duraphat, Colgate Palmolive Co.) at D90 (Fig. 1).

### Procedures

To ensure blinding and reduce methodological bias, both subject’s study teeth received pre-treatment as recommended for SAP P_11_-4: dental cleaning with pumice stone (incl. 2% NaOCl, Districhemie SA, Ecublens, Switzerland) and etching (Etching Gel, Ultra-etch, Ultradent, UT, USA 35% H_3_PO_4_, 20 s). Treatment was provided on D0, and all study teeth received fluoride varnish at D90. Additional study visits were D30, D180 and D270. Standardized clinical photographs including a grey-scale in every picture for calibration were taken^15,36–39^, oral hygiene, plaque index (according to Silness and Loe 1964^40^), Nyvad Caries Activity Criteria^35^ and laser fluorescence reading (Diagnodent Pen; KaVo) recorded as well as oral hygiene instructions provided at every study visit.

### Outcomes

**Figure 2:**
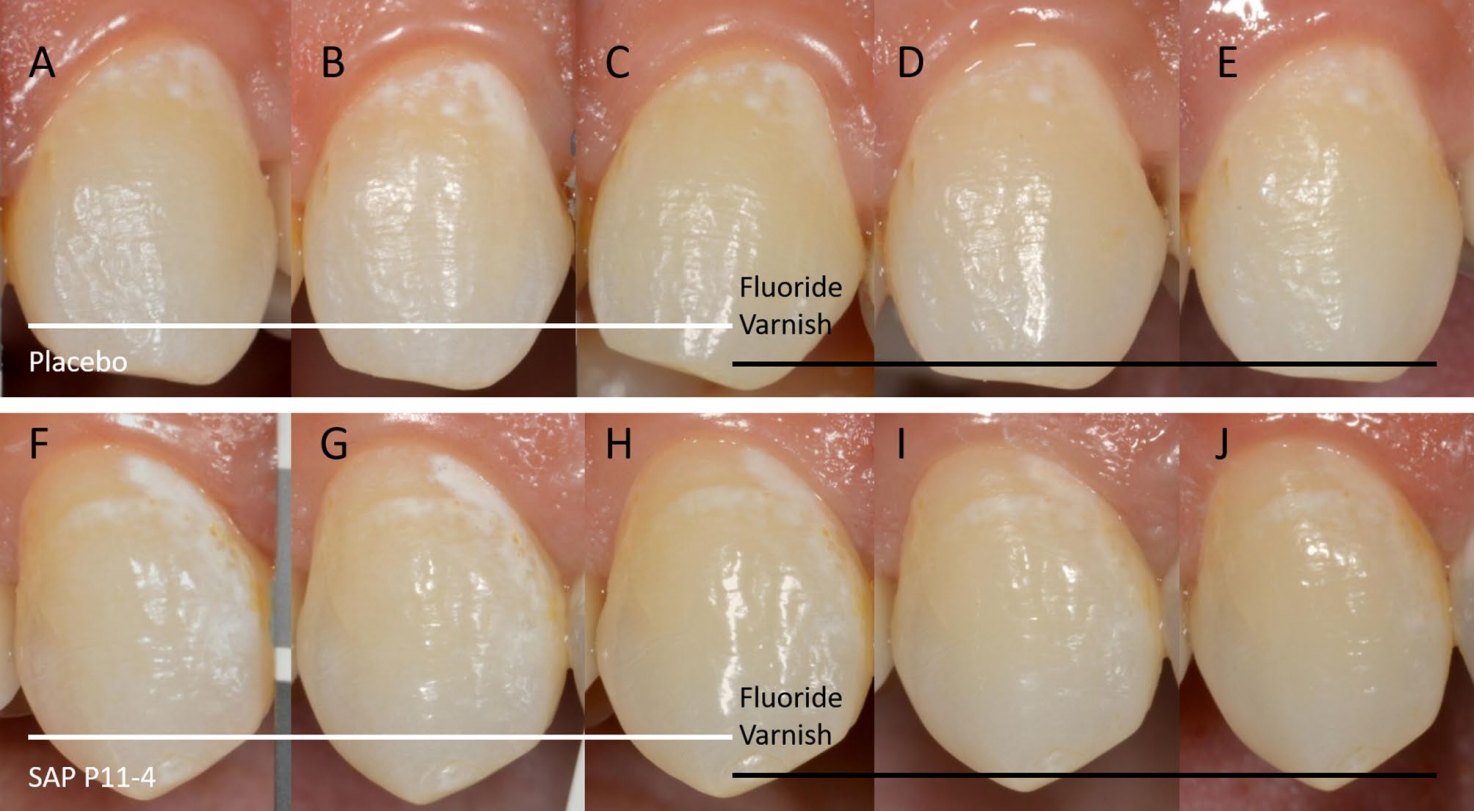
Clinical standardized photographs of one subject, showing the control lesion (**A**–**E**) and the test lesion (**F**–**J**) at each interval: Day 0 (**A**, **F**); Day 30 (**B**, **G**); Day 90 (**C**, **H**); Day 180 (**D**, **I**); Day 270 (**E**, **I**). The treatments and their time periods within the clinical design are indicated at the bottom of the photographs.

The primary outcome was the regression/progression of white spot lesions as judged by the change in WSL size assessed by morphometry^15,20,21,23,41^ on standardized, calibrated clinical photographs as described in Broeseler et al.^23^ (Fig. 2). Morphometric assessment was performed by a blinded assessor (Image J, Version 1.45, NIH, Bethesda, MA, USA) and measurements were reviewed by another blinded person for plausibility and for consistency of morphometric measurements over time (March 2016–October 2018).

The secondary parameters were: lesion’s activity assessed by Nyvad Caries Activity Criteria, Global Impression of Change Questionnaire (GIOCQ), Laser Fluorescence value readings and Visual Analogue Scale (VAS) for the progression/regression of each lesion with respect to baseline as judged by the clinician at each visit. Due to the non-linearity of VAS, only meaningful study groups starting at D0 were assessed (same argument is valid also for GIOCQ and Nyvad Caries Activity Criteria). The VAS scale ranged from − 50 to + 50, with negative values indicating regression and “0” arrested and positive values progression. Safety of treatments was assessed by recording adverse events per Directive 2001/20/EC, ISO 14155 and MDD93/42.

### Statistical analysis

The changes in WSL size between the test and control arm were evaluated using Hierarchical Linear Modelling (HLM, two-sided), which allowed to integrate important variables into the model, to account for confounding factors and to evaluate the effect of treatment, taking into account correlations resulting from the paired measurements. The dependent variable of the HLM models was the morphometric change in WSL size due to the treatments over time from D0, whilst the independent variables were: gender (dummy variable with “female” as a baseline), race (converted in a dummy variable with “Caucasian” as a baseline), age, DMFT, WSL clinical index, oral hygiene and plaque index, with all explanatory variables adjusted for their respective baseline values. The effect of the treatment(s) was evaluated using a dummy variable with the control group as baseline. The following four group comparisons (corresponding to the four Hypothesis H1–H4) were of interest in the present study:

- (H1) Placebo vs SAP P_11_-4 (D90–D0 of the control arm vs. D90–D0 of the test arm).
- (H2) Fluoride varnish (FV) vs SAP P_11_-4 (D180–D90 of the control arm vs. D90–D0 of the test arm).
- (H3) FV vs SAP P_11_-4 + delayed FV (D270–D90 of the control arm vs. D180–D0 of the test arm) to compare FV with a combined treatment of SAP P_11_-4 and FV in a period of 180 days.
- (H4) FV vs FV with prior SAP P_11_-4 application (D270–D90 of the control arm vs. D270–D90 of the test arm) to investigate whether prior application of SAP P_11_-4 had an influence on FV.

The null hypothesis for each of the four comparisons of interest was that there was no difference in the morphometric change between each comparison, while the alternative hypothesis was that there was a difference. One HLM fixed effects model for each of the four hypotheses above was estimated for treatment and control variables and the associated statistics presented. The assumptions for the HLM models (normality and homoscedasticity) have been tested and hold for the four hypotheses. The effect of treatment was considered statistically significant if the p-value of the treatment dummy variable was smaller than 0.01. All analyses were run in IBM SPSS Statistics Version 24 (January 2019–June 2019). Intention-to-treat analysis was performed.

Sensitivity analysis of HLM was performed varying the inclusion of parameters in the model as well as computed parameter values themselves (average oral hygiene or plaque index value of the two investigated time points for each group during a comparison and their respective value at the start time of the comparison interval in both arms) in order to investigate the robustness of the model or the result.

At study start, the available literature did not yield suitable data for a sample size calculation. Therefore, the estimation for the primary outcome based on successful alike studies^42^. A post hoc calculation of the sample size for a 0.8 power level and 0.01 significance level for the three comparisons (H1–H3) ranged between 42 and 51 pairs of observations. Sample size calculations were computed using STATA 15.1 by StataCorp (College Station, TX, USA) "Power and Sample size" module.

All secondary parameters were analysed descriptively, since the estimation of the sample size was based only on the primary parameter.

## Supplementary information

Supplementary Information.

## Data Availability

The datasets generated during and analysed during the current study are available from the corresponding author on request.
