# Supplementary material for: Efficacy of P11-4 for the treatment of initial buccal caries: a randomized clinical trial
Source: Sci Rep. 2020 Nov 19;10:20211. doi: 10.1038/s41598-020-77057-3 (PMC7678830; doi:10.1038/s41598-020-77057-3)
Supplement: Supplementary file 1 — Supplementary Information. [file 41598_2020_77057_MOESM1_ESM.pdf]

SUPPLEMENT MATERIAL

Scientific Reports

Efficacy of P_{11}-4 for the treatment of initial buccal caries: A randomized clinical trial

Paulina Sedlakova Kondelova[1#], Alaa Mannaa[2#], Claudine Bommer[3], Marwa Abdelaziz[1], Laurent Daeniker[1], Enrico di Bella[4], Ivo Krejci[1*]

Supplementary Figures

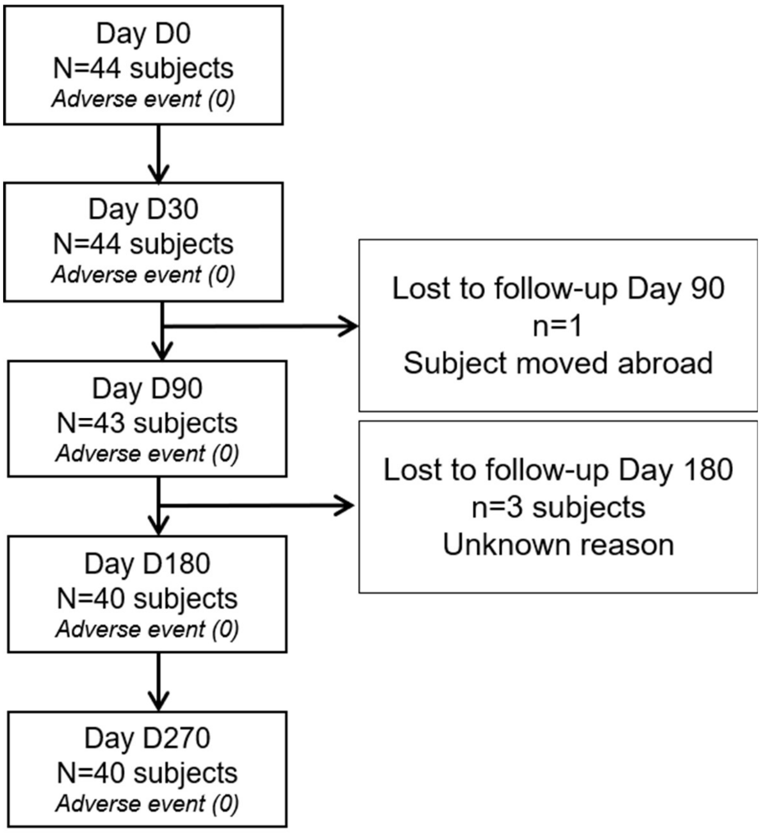

Supplementary Figure S1: Consort Chart. Subject flow throughout the trial. Due to the split-mouth design of the clinical trial, only subjects are indicated.

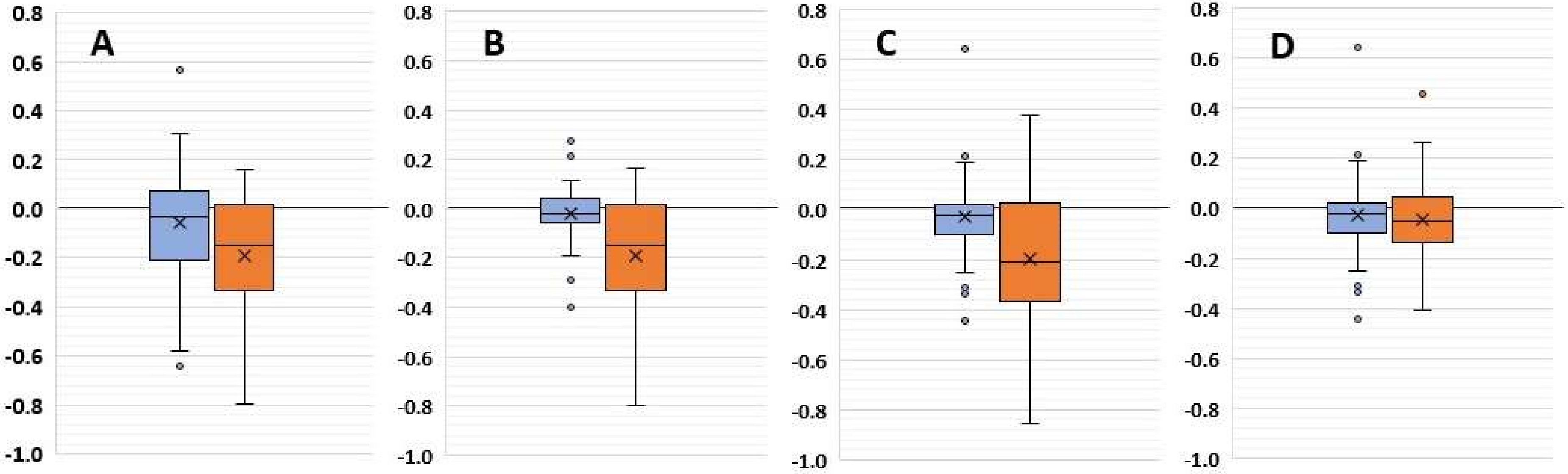

Supplementary Figure S2: Box plots of the four group comparisons showing the change in white spot lesions size assessed by morphometry for the test and control group. (A) Placebo vs SAP P11-4 (H1, D90-D0 of the control arm vs. D90-D0 of the test arm), (B) Fluoride varnish (FV) vs SAP P11-4 (H2, D180-D90 of the control arm vs. D90-D0 of the test arm), (C) FV vs SAP P11-4 + delayed FV (H3, D270-D90 of the control arm vs. D180-D0 of the test arm), (D) FV vs FV with prior SAP P11-4 application (H4, D270-D90 of the control arm vs. D270-D90 of the test arm).

Supplementary Tables

Supplementary Table S1: Relative lesion size of the morphometric measurements with respect to baseline.

Group	Morphometry	D0	D30	D90	D180	D270
Control	Mean	1.00	0.98	0.96	0.92	0.90
	Std. dev.	0.00	0.21	0.27	0.28	0.32
	valid cases	44	44	43	40	39
	Min	1.00	0.45	0.36	0.31	0.17
	1Q	1.00	0.90	0.79	0.79	0.76
	Median	1.00	1.01	0.96	0.97	0.97
	3Q	1.00	1.08	1.07	1.09	1.07
	Max	1.00	1.46	1.89	1.42	1.60
Test	Mean	1.00	0.91	0.81	0.80	0.76
	Std. dev.	0.00	0.21	0.26	0.28	0.30
	valid cases	43	42	41	39	39
	Min	1.00	0.25	0.21	0.15	0.18
	1Q	1.00	0.84	0.70	0.63	0.55
	Median	1.00	0.95	0.87	0.79	0.71
	3Q	1.00	0.99	1.02	1.02	1.01
	Max	1.00	1.45	1.16	1.38	1.32

Supplementary Table S2: Results of the hierarchical linear modelling for the group comparison Placebo vs SAP P11-4 (H1, D90-D0 of the control arm vs. D90-D0 of the test arm) regarding the treatment effect assessed on the basis of morphometry.

Estimates of Fixed Effects[a]

						95% Confidence Interval	
Parameter	Estimate	Std. Error	df	t	Sig.	Lower Bound	Upper Bound
Intercept	.426512	.287661	65	1.483	.143	-.147986	1.001010
Sex = Male	.081582	.061322	65	1.330	.188	-.040887	.204051
Race = Non-Caucasian	.137757	.065021	65	2.119	.038	.007900	.267613
Age	-.002629	.007679	65	-.342	.733	-.017965	.012706
DMFT	.018299	.007291	65	2.510	.015	.003738	.032860
Treatment	-.138340	.050386	65	-2.746	.008	-.238969	-.037711
Clinical Index WSL	.027381	.034786	65	.787	.434	-.042092	.096854
Oral Hygiene D0	-.128465	.032320	65	-3.975	.000	-.193012	-.063917
Plaque Index D0	-.382319	.121487	65	-3.147	.002	-.624945	-.139693

a. Dependent Variable: H1 Test D90-D0 vs Control D90-D0.

Supplementary Table S3: Results of the hierarchical linear modelling for the group comparison Fluoride varnish (FV) vs SAP P11-4 (H2, D180-D90 of the control arm vs. D90-D0 of the test arm) regarding the treatment effect assessed on the basis of morphometry.

Estimates of Fixed Effects[a]

Parameter	Estimate	Std. Error	df	t	Sig.	95% Confidence Interval Lower Bound	95% Confidence Interval Upper Bound
Intercept	-.014461	.264467	65	-.055	.957	-.542638	.513716
Sex = Male	.044231	.056378	65	.785	.436	-.068363	.156826
Race = Non-Caucasian	.059350	.059779	65	.993	.324	-.060036	.178736
Age	.007064	.007059	65	1.001	.321	-.007035	.021163
DMFT	.001685	.006703	65	.251	.802	-.011702	.015072
Treatment	-.176062	.046324	65	-3.801	.000	-.268577	-.083547
Clinical Index WSL	.027166	.031982	65	.849	.399	-.036706	.091037
Oral Hygiene D0	-.064259	.029714	65	-2.163	.034	-.123602	-.004916
Plaque Index D0	-.234202	.111691	65	-2.097	.040	-.457266	-.011139

a. Dependent Variable: H2 Test D90-D0 vs Control D180-D90.

Supplementary Table S4: Results of the hierarchical linear modelling for the group comparison FV vs SAP P11-4 + delayed FV (H3, D270-D90 of the control arm vs. D180-D0 of the test arm) regarding the treatment effect assessed on the basis of morphometry.

Estimates of Fixed Effects[a]

Parameter	Estimate	Std. Error	df	t	Sig.	95% Confidence Interval Lower Bound	95% Confidence Interval Upper Bound
Intercept	.014915	.312753	65	.048	.962	-.609695	.639525
Sex = Male	.038525	.066671	65	.578	.565	-.094627	.171676
Race = Non-Caucasian	.116935	.070693	65	1.654	.103	-.024249	.258118
Age	.004540	.008348	65	.544	.588	-.012133	.021213
DMFT	.003200	.007927	65	.404	.688	-.012632	.019031
Treatment	-.168259	.054782	65	-3.071	.003	-.277665	-.058853
Clinical Index WSL	.022036	.037821	65	.583	.562	-.053497	.097569
Oral Hygiene D0	-.052709	.035139	65	-1.500	.138	-.122887	.017468
Plaque Index D0	-.322164	.132084	65	-2.439	.017	-.585954	-.058375

a. Dependent Variable: H3 Test D180-D0 vs Control D270-D90.

Supplementary Table S5: Results of the hierarchical linear modelling for the group comparison FV vs FV with prior SAP P11-4 application (D, D270-D90 of the control arm vs. D270-D90 of the test arm) regarding the treatment effect assessed on the basis of morphometry.

Estimates of Fixed Effects[a]

Parameter	Estimate	Std. Error	df	t	Sig.	95% Confidence Interval	
						Lower Bound	Upper Bound
Intercept	-.220182	.227948	65	-.966	.338	-.675426	.235061
Sex = Male	.025828	.048593	65	.532	.597	-.071219	.122875
Race = Non-Caucasian	.017972	.051524	65	.349	.728	-.084929	.120873
Age	.004048	.006085	65	.665	.508	-.008104	.016200
DMFT	-.006450	.005778	65	-1.116	.268	-.017989	.005089
Treatment	-.015650	.039927	65	-.392	.696	-.095390	.064090
Clinical Index WSL	.015495	.027565	65	.562	.576	-.039557	.070547
Oral Hygiene D0	.015040	.025611	65	.587	.559	-.036109	.066189
Plaque Index D0	-.111923	.096269	65	-1.163	.249	-.304185	.080338

a. Dependent Variable: H4 Test D270-D90 vs Control D270-D90.

Supplementary Table S6: HLM results of sensitivity analysis using the average of plaque index and oral hygiene for the group comparison Placebo vs. SAP P11-4 (H1, D90-D0 of the control arm vs. D90-D0 of the test arm) in relation to the treatment effect evaluated on the basis of morphometry.

Estimates of Fixed Effects[a]

Parameter	Estimate	Std. Error	df	t	Sig.	95% Confidence Interval	
						Lower Bound	Upper Bound
Intercept	.833173	.382732	65	2.177	.033	.068805	1.597541
Sex = Male	.083677	.063150	65	1.325	.190	-.042443	.209797
Race = Non-Caucasian	.121664	.066139	65	1.840	.070	-.010424	.253751
Age	-.004007	.008641	65	-.464	.644	-.021264	.013250
DMFT	.014575	.007528	65	1.936	.057	-.000460	.029610
Treatment	-.136852	.051643	65	-2.650	.010	-.239991	-.033714
Clinical Index WSL	.016373	.035835	65	.457	.649	-.055194	.087941
OH Average	-.195735	.052303	65	-3.742	.000	-.300192	-.091279
PI Average	-.412362	.173407	65	-2.378	.020	-.758680	-.066044

a. Dependent Variable: H1 Test D90-D0 vs Control D90-D0.

Supplementary Table S7: HLM results of sensitivity analysis using the average of plaque index and oral hygiene for the group comparison Fluoride varnish (FV) vs SAP P11-4 (H2, D180-D90 of the control arm vs. D90-D0 of the test arm) in relation to the treatment effect evaluated on the basis of morphometry.

Estimates of Fixed Effects[a]

Parameter	Estimate	Std. Error	df	t	Sig.	95% Confidence Interval	
						Lower Bound	Upper Bound
Intercept	.449854	.335199	65	1.342	.184	-.219584	1.119292
Sex = Male	.040640	.055307	65	.735	.465	-.069817	.151096
Race = Non-Caucasian	.061759	.057924	65	1.066	.290	-.053924	.177442
Age	.002475	.007568	65	.327	.745	-.012639	.017588
DMFT	.001623	.006593	65	.246	.806	-.011545	.014790
Treatment	-.175372	.045230	65	-3.877	.000	-.265702	-.085043
Clinical Index WSL	.022063	.031384	65	.703	.485	-.040616	.084742
OH Average	-.126889	.045807	65	-2.770	.007	-.218373	-.035405
PI Average	-.426094	.151871	65	-2.806	.007	-.729401	-.122787

a. Dependent Variable: H2 Test D90-D0 vs Control D180-D90.

Supplementary Table S8: HLM results of sensitivity analysis using the respective Plaque Index and Oral Hygiene values at the beginning of the comparison interval for the group comparison Fluoride varnish (FV) vs SAP P11-4 (H2, D180-D90 of the control arm vs. D90-D0 of the test arm) in relation to the treatment effect evaluated on the basis of morphometry.

Estimates of Fixed Effects[a]

						95% Confidence Interval	
Parameter	Estimate	Std. Error	df	t	Sig.	Lower Bound	Upper Bound
Intercept	.433945	.335670	65	1.293	.201	-.236435	1.104325
Sex = Male	.020038	.056067	65	.357	.722	-.091935	.132011
Race = Non-Caucasian	.039739	.056931	65	.698	.488	-.073961	.153439
Age	.000112	.008100	65	.014	.989	-.016065	.016289
DMFT	-.000179	.006440	65	-.028	.978	-.013041	.012683
Treatment	-.207560	.046405	65	-4.473	.000	-.300237	-.114883
Clinical Index WSL	.015147	.031419	65	.482	.631	-.047601	.077895
OH start time	-.102126	.034865	65	-2.929	.005	-.171756	-.032496
PI start time	-.300525	.123409	65	-2.435	.018	-.546990	-.054061

a. Dependent Variable: H2 Test D90-D0 vs Control D180-D90.

Supplementary Table S9: HLM results of sensitivity analysis using the average of plaque index and oral hygiene for the group comparison FV vs SAP P11-4 + delayed FV (H3, D270-D90 of the control arm vs. D180-D0 of the test arm) in relation to the treatment effect evaluated on the basis of morphometry.

Estimates of Fixed Effects[a]

						95% Confidence Interval	
Parameter	Estimate	Std. Error	df	t	Sig.	Lower Bound	Upper Bound
Intercept	.132381	.345137	65	.384	.703	-.556906	.821668
Sex = Male	.055031	.068449	65	.804	.424	-.081672	.191733
Race = Non-Caucasian	.121045	.071815	65	1.686	.097	-.022379	.264468
Age	.005699	.008504	65	.670	.505	-.011284	.022682
DMFT	.003103	.007982	65	.389	.699	-.012838	.019044
Treatment	-.167460	.054976	65	-3.046	.003	-.277254	-.057666
Clinical Index WSL	.016125	.038053	65	.424	.673	-.059872	.092122
OH Average	-.077834	.048545	65	-1.603	.114	-.174786	.019117
PI Average	-.549517	.231619	65	-2.373	.021	-1.012093	-.086942

a. Dependent Variable: H3 Test D180-D0 vs Control D270-D90.

Supplementary Table S10: HLM results of sensitivity analysis using the respective Plaque Index and Oral Hygiene values at the beginning of the comparison interval for the group comparison FV vs SAP P11-4 + delayed FV (H3, D270-D90 of the control arm vs. D180-D0 of the test arm) in relation to the treatment effect evaluated on the basis of morphometry.

Estimates of Fixed Effects[a]

						95% Confidence Interval	
Parameter	Estimate	Std. Error	df	t	Sig.	Lower Bound	Upper Bound
Intercept	.547597	.399292	65	1.371	.175	-.249843	1.345038
Sex = Male	-.006367	.066693	65	-.095	.924	-.139563	.126829
Race = Non-Caucasian	.094576	.067722	65	1.397	.167	-.040674	.229826
Age	-.007589	.009635	65	-.788	.434	-.026831	.011654
DMFT	.001938	.007661	65	.253	.801	-.013362	.017238
Treatment	-.192150	.055200	65	-3.481	.001	-.302393	-.081908
Clinical Index WSL	.012334	.037374	65	.330	.742	-.062307	.086976
OH start point	-.077707	.041473	65	-1.874	.065	-.160534	.005120
PI start point	-.431469	.146799	65	-2.939	.005	-.724648	-.138291

a. Dependent Variable: H3 Test D180-D0 vs Control D270-D90.

Supplementary Table S11: HLM results of sensitivity analysis using the average of plaque index and oral hygiene for the group comparison FV vs FV with prior SAP P11-4 application (H4, D270-D90 of the control arm vs. D270-D90 of the test arm) in relation to the treatment effect evaluated on the basis of morphometry.

Estimates of Fixed Effects[a]

						95% Confidence Interval	
Parameter	Estimate	Std. Error	df	t	Sig.	Lower Bound	Upper Bound
Intercept	.490593	.346179	65	1.417	.161	-.200773	1.181959
Sex = Male	.015609	.046966	65	.332	.741	-.078189	.109406
Race = Non-Caucasian	.024534	.048243	65	.509	.613	-.071813	.120881
Age	-.006500	.007001	65	-.928	.357	-.020482	.007483
DMFT	-.001803	.005872	65	-.307	.760	-.013531	.009925
Treatment	-.012862	.037829	65	-.340	.735	-.088411	.062688
Clinical Index WSL	-.005138	.026919	65	-.191	.849	-.058899	.048623
OH Average	-.052726	.045224	65	-1.166	.248	-.143044	.037591
PI Average	-.541162	.181196	65	-2.987	.004	-.903036	-.179288

a. Dependent Variable: H4 Test D270-D90 vs Control D270-D90.

Supplementary Table S12: HLM results of sensitivity analysis using the respective Plaque Index and Oral Hygiene values at the beginning of the comparison interval for the group comparison FV vs FV with prior SAP P11-4 application (H4, D270-D90 of the control arm vs. D270-D90 of the test arm) in relation to the treatment effect evaluated on the basis of morphometry.

Estimates of Fixed Effects[a]

Parameter	Estimate	Std. Error	df	t	Sig.	95% Confidence Interval	
						Lower Bound	Upper Bound
Intercept	.428683	.305251	65	1.404	.165	-.180946	1.038312
Sex = Male	.001055	.046973	65	.022	.982	-.092756	.094866
Race = Non-Caucasian	.011774	.047222	65	.249	.804	-.082534	.106083
Age	-.005256	.006738	65	-.780	.438	-.018712	.008201
DMFT	-.006334	.005403	65	-1.172	.245	-.017124	.004456
Treatment	-.014696	.037630	65	-.391	.697	-.089849	.060457
Clinical Index WSL	.008438	.026043	65	.324	.747	-.043573	.060449
OH start time	-.055420	.039032	65	-1.420	.160	-.133373	.022532
PI start time	-.366504	.110452	65	-3.318	.001	-.587091	-.145917

a. Dependent Variable: H4 Test D270-D90 vs Control D270-D90.

Supplementary Table S13: Absolute laser fluorescence values.

Group	Laser fluorescence	D0	D30	D90	D180	D270
**Control**	Mean	6.5	6.3	6.5	6.4	6.4
	Std. dev.	4.9	3.4	4.6	5.7	5.2
	valid cases	44	44	43	40	40
**Test**	Mean	6.7	6.9	6.7	7.1	6.8
	Std. dev.	5.3	5.7	4.5	7.0	5.7
	valid cases	44	44	43	40	40

Supplementary Table S14: Active and inactive lesions according to Nyvad Caries Activity Criteria (n).

Group	Nyvad Criteria	D0	D30	D90	D180	D270
**Control**	Active (1, 2)	30	27	20	17	17
	Inactive (4, 5)	14	17	23	23	23
	Missing data	0	0	0	0	0
**Test**	Active (1, 2)	25	22	17	15	10
	Inactive (4, 5)	18	21	25	24	29
	Missing data	1	1	1	1	1
	Lost to follow-up	0	0	1	4	4

Supplementary Table S15: Oral hygiene (n).

**Oral Hygiene**						
**Code**	**Description**	**D0**	**D30**	**D90**	**D180**	**D290**
1	poor	0	0	0	0	1
2	unsatisfactory	3	1	1	1	1
3	sufficient	5	4	2	4	3
4	good	11	9	11	9	12
5	excellent	25	30	29	26	23
	Lost to follow-up	0	0	1	4	4
	Available	44	44	43	40	40

Supplementary Table S16: Plaque index (PI).

PI	D0	D30	D90	D180	D290
Mean	0.20	0.13	0.16	0.12	0.16
Std. Dev.	0.25	0.16	0.26	0.13	0.20
